# The Story of the Insane from Year to Year

**Published:** 1897-09-25

**Authors:** 


### THE STORY OF THE INSANE FROM YEAR TO YEAR.

Tenth Series.

### NORWICH CITY ASYLUM.

On January 1st, 1896, this asylum contained 313 patients, and by the end of the year the numbers had fallen to 296. There were 81 admissions, 19 readmissions, 36 recoveries, 36 discharged relieved, and 32 deaths. The recoveries, calculated on the admissions, stand at 39·13 per cent., and the deaths, calculated on the average number resident, stand at 10·49. Of the deaths 23 were caused by cerebral and spinal diseases, and 5 by consumption. Of the total number of admissions 13 were driven insane by such causes as domestic trouble and anxiety, 20 by intemperance in drink, and 29 by hereditary influence. Dr. Harris is one of those conscientious medical superintendents who decline to make post-mortems without the consent of the relatives of the deceased, and yet he was able to get this consent in 53 per cent. of the deaths. More than this can never be necessary. The slight decrease in the numbers which we have noted was caused by the removal of some out-county patients and not by any decrease in the home cases. Indeed, we find that at the commencement of the present decade there were only 181 Norwich patients in the asylum, and now there are 257, showing an annual increase of eight, which means that in six years the asylum will be full. As the enlargement of the asylum is inevitable, it would surely be wise to begin the additions while breathing time remains, and not postpone, as is so often done, that evil day of bricks and mortar which everyone connected with asylums dreads, but which everyone has to face. We have ere now met with medical superintendents who would gladly dispense with their pulpits; but Dr. Harris is the only one we ever heard of who invented a pulpit, albeit his design would seem to be really good. The visitors say they are happy in retaining the services of their medical superintendent. The weekly cost of maintenance is a fraction under 9s.

